# PIC-Me: paralogs and isoforms classifier based on machine-learning approaches

**DOI:** 10.1186/s12859-021-04229-x

**Published:** 2021-10-21

**Authors:** Jooseong Oh, Sung-Gwon Lee, Chungoo Park

**Affiliations:** School of Biological Sciences and Technology, Chonnam National University, Gwangju, 61186 Republic of Korea

**Keywords:** Gene duplication, Paralogs, Alternative splicing, Isoforms, Machine learning, RNA-Seq

## Abstract

**Background:**

Paralogs formed through gene duplication and isoforms formed through alternative splicing have been important processes for increasing protein diversity and maintaining cellular homeostasis. Despite their recognized importance and the advent of large-scale genomic and transcriptomic analyses, paradoxically, accurate annotations of all gene loci to allow the identification of paralogs and isoforms remain surprisingly incomplete. In particular, the global analysis of the transcriptome of a non-model organism for which there is no reference genome is especially challenging.

**Results:**

To reliably discriminate between the paralogs and isoforms in RNA-seq data, we redefined the pre-existing sequence features (sequence similarity, inverse count of consecutive identical or non-identical blocks, and match-mismatch fraction) previously derived from full-length cDNAs and EST sequences and described newly discovered genomic and transcriptomic features (twilight zone of protein sequence alignment and expression level difference). In addition, the effectiveness and relevance of the proposed features were verified with two widely used support vector machine (SVM) and random forest (RF) models. From nine RNA-seq datasets, all AUC (area under the curve) scores of ROC (receiver operating characteristic) curves were over 0.9 in the RF model and significantly higher than those in the SVM model.

**Conclusions:**

In this study, using an RF model with five proposed RNA-seq features, we implemented our method called Paralogs and Isoforms Classifier based on Machine-learning approaches (PIC-Me) and showed that it outperformed an existing method. Finally, we envision that our tool will be a valuable computational resource for the genomics community to help with gene annotation and will aid in comparative transcriptomics and evolutionary genomics studies, especially those on non-model organisms.

**Supplementary Information:**

The online version contains supplementary material available at 10.1186/s12859-021-04229-x.

## Background

Gene duplication and alternative splicing have played central roles in defining protein diversity and its link to phenotypic variations. Gene duplication that gives rise to the production of two genes encoding distinct proteins with different functions is a phenomenon that occurs as a result of a number of dynamic cellular events, including chromosomal (or genome) duplications, retroposition, or unequal crossing over (reviewed in [1–3]). After duplication, from a functional redundancy perspective, the predominant fate of duplicates is pseudogenization; however, a significant fraction of duplicated genes (hereafter called paralogs) is preserved by either subfunctionalization or neofunctionalization. This event plays a fundamental role in the evolution of genomes and organisms. Alternative splicing is a post-transcriptional process that generates multiple mRNAs from the same precursor-mRNA and plays a critical role in cell development, physiological processes, and various diseases (reviewed in [4–6]). It is now firmly established that this alternative splicing event is prevalent in all multicellular eukaryotes.

Intriguingly, these two disparate events are closely linked. Multiple transcript isoforms generated by alternative splicing (hereafter called isoforms) especially via mutually exclusive exons, where an exclusive exon is selected from two or more exons in the pre-mRNA, are considered to have an “internal paralog” in the same gene [7]. These isoforms have novel functions that can evolve without disrupting the original function of the gene, and this scenario is compatible with the neofunctionalization model (that is, the gain of a new function by a duplicate gene [8]) of duplicate gene evolution. Because of this reason and the growing interest in this phenomenon, evolutionary and functional analyses of gene duplication and alternative splicing events have become a popular topic in the evolutionary genomics field.

To explain this relationship between gene duplication and alternative splicing, three theoretical models (independent, functional sharing, and accelerated alternative splicing models; depicted in Fig. 2 in [9]) have been proposed [10], and several studies have presented analytical fits to experimental data. For example, in comparison with non-duplicated single-copy genes, larger gene families that originated from duplication events have fewer genes affected by alternative splicing events, and the number of alternative splicing events per gene is lower in larger gene families. These results supporting the functional sharing model have been observed in humans, mice, and worms [11–13] but not in plants [14]. Roux and Robinson-Rechavi [15] additionally reported a positive correlation between the number of alternative splicing events and the evolutionary time after gene duplication and found that paralogs under higher purifying selection have a lower rate of acquisition of new splicing forms. Furthermore, two independent studies showed that paralogs that experienced an alternative splicing event had higher expression variation than those that did not experience such events [16, 17].

Despite their recognized importance and the advent of large-scale genomic and transcriptomic analyses, paradoxically, obtaining an accurate annotation of each gene locus to identify paralogs and isoforms remains surprisingly difficult. This difficulty is mainly due to the lack of completely assembled genomes and the difficulty of assembling and obtaining full-length transcripts [18]. Recently, Spitzer et al. [19] studied genetic factors that can be used to facilitate the discrimination between paralogs and isoforms. They proposed three sequence-alignment-based features and developed a machine learning classifier to distinguish between paralogs and isoforms without the need to access the genomic data, including the reference genome and annotation information. However, this approach requires substantial conceptual and methodological improvements when full-length cDNAs and EST sequences are unavailable. Indeed, recent advances in transcriptome analysis facilitated by RNA sequencing (RNA-seq) technology make it possible to characterize and annotate the transcriptome. However, it is still not clear, and may never be, how exactly full-length transcripts can be reconstructed when used de novo.

In this study, to reliably identify and classify the paralogs and isoforms in RNA-seq data, we redefined the pre-existing sequence features of possibly fragmented and misassembled transcripts and described newly discovered genomic and transcriptomic features. Using a random forest (RF) model with all of the suggested RNA-seq features, we implemented our new tool, *P*aralogs and *I*soforms *C*lassifier based on *M*achine-l*e*arning approaches (PIC-Me), and showed that our method outperformed an existing method.

## Methods

### Data collection

We collected publicly available RNA-seq data for three animal tissues (brain, ovary, and testis) from humans [20] and zebrafish [21] and three plant seed tissues (aleurone layer, transfer cells, and whole endosperm) from wheat [22].

### Transcriptome analysis

To discard low-quality reads and trim the adaptor sequences, all nine RNA-seq datasets were preprocessed using Trimmomatic v.0.36 [23]. Using our stepwise transcriptome assembly pipeline [24], we reconstructed all transcripts from each tissue in each species. Briefly, the trimmed read sequences were separately de novo assembled for each tissue using Trinity v.2.2.0 [25] with the default parameters. The coding sequences (CDSs) within the assembled transcripts were predicted using TransDecoder v.3.0.0 (https://github.com/TransDecoder/TransDecoder) aided by BLASTP searches [26] in the Uniprot/Swiss-Prot database [27] with an *E*-value cutoff 10^–5^. To obtain high-quality non-redundant transcripts, those with a CDS length < 100 amino acids or 99% sequence identity were removed. To quantify the expression level of each transcript, the RNA-seq reads from each sample were mapped to the corresponding non-redundant transcriptome database using bowtie (v.2.2.6) [28], and their expression levels were estimated with RSEM (v.1.2.26) [29]. The unit of expression level in our analysis is referred to as fragment per kilobase of transcript per million fragments mapped (FPKM).

### Annotation between paralogs and isoforms

We obtained the lists of paralogous gene pairs from the Ensembl Compara homology database (version 95) [30] via biomart. Using the single linkage clustering method, these gene pairs were clustered into gene families. To obtain the isoforms at a transcript sequence level, gene data, including gene description and location, were collected from biomart, and transcripts were designated as isoforms if they had the same ENSG ID but a different ENST ID. To annotate the assembled transcripts, we performed BLASTP searches against the human (GRCh38), zebrafish (GRCz11), and wheat (IWGSC RefSeq v1.0) protein databases from Ensembl (https://www.ensembl.org) and EnsemblPlants (https://plants.ensembl.org) with an *E*-value cutoff 10^–10^.

### Bioinformatic features for the classification of paralogs versus isoforms

**Fig. 1:**
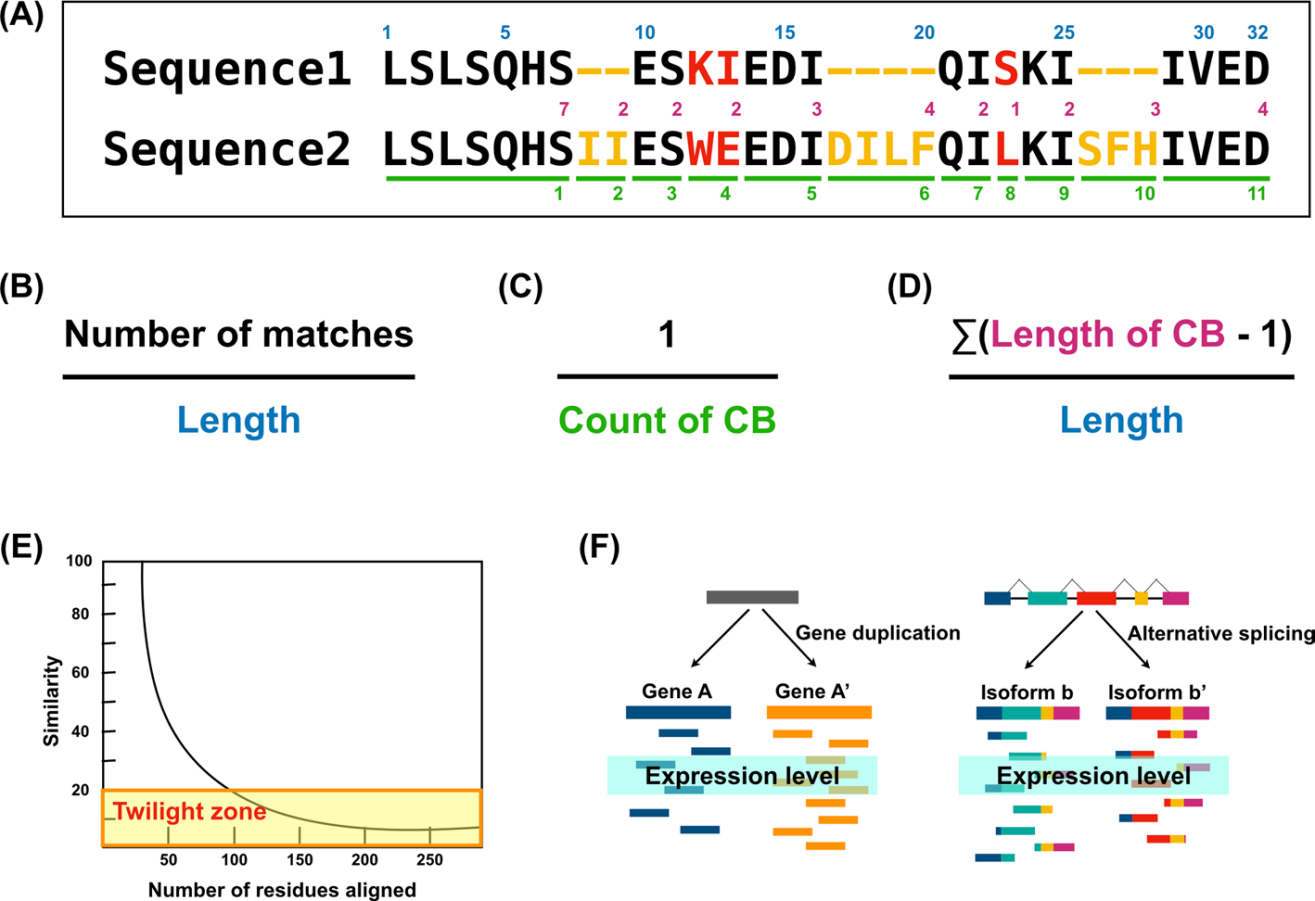
Illustration of the five features. **A** Two amino acid sequences (sequence1 and sequence2) are aligned. Matches, mismatches, and gaps between the two sequences are colored in black, red, and yellow, respectively. Green underlining indicates the consecutive identical or non-identical blocks (CB). **B** Sequence similarity (SS) is the percentage of matched sequences in the aligned sequences. **C** Inverse count of consecutive identical or non-identical blocks (ICCB) is the inverse count of CB. **D** Match-mismatch fraction (MMF) indicates the overall number of consecutive matches and mismatches–namely, it is the sum of the sequence lengths minus one of all CB divided by the length of the alignment. **E** Twilight zone (TZ) is a range of sequence similarity; a 20% cut-off score was used here. **F** Expression level difference (ELD) is the difference in the expression levels between two genes. From the example alignment in **A**, the scores of the first three features were 0.625 for SS, 0.091 for ICCB, and 0.656 for MMF, which are detailly described in Additional file 1: Fig. S1

To discriminate between paralogs and isoforms, we considered five genomic and transcriptomic features, including sequence similarity (SS), inverse count of consecutive identical or non-identical blocks (ICCB), match-mismatch fraction (MMF), twilight zone of protein sequence alignment (TZ), and expression level difference (ELD). The first three genomic features were adopted from the study by Spitzer et al. [19]; these were used for full-length cDNA and EST sequences from a public database, not with RNA-seq data, to distinguish between isoforms and paralogs. For our RNA-seq-based analysis, we used the same concepts and definitions. Briefly, SS is the fraction of the number of matches in the alignment of the sequences. ICCB is the reciprocal value of the number of blocks in which the alignments are consecutively matched or mismatched. MMF represents the normalized number of consecutive matches and mismatches–namely, the sum of the lengths minus one of all consecutive identical or non-identical blocks divided by the length of alignment. Next, TZ, which has been defined as the range of sequence length with 20–35% sequence identity that can unambiguously distinguish between protein pairs with similar and non-similar functions [31], was used as a cut-off value. In this study, pairs of proteins with less than 20% SS were excluded because they could not be correctly identified as paralogs or isoforms. Finally, ELD is the log-transformed absolute value of the FPKM difference between two transcripts. These features are schematically illustrated in Fig. 1 and Additional file 1: Fig. S1.

The pairs of sequences were aligned using the program *fftnsi* in the MAFFT package [32] with default parameters.

### Machine learning models

We trained and applied two supervised machine learning algorithms: support vector machine (SVM) and random forest (RF). For the SVM classifier, we used the SVM^light^ (http://svmlight.joachims.org) package [33], consisting of two modules: svm_learn and svm_classify. The former module is used to learn input–output functionality from the training dataset (positively and negatively labeled for paralogs and isoforms, respectively), and the latter is used to classify the data by using the models prepared by svm_learn. In this study, the radial basis function (RBF) was used as a kernel function for the SVM classifier. Except for C and g describing the trade-off between training error and margin and the width of the Gaussian bells, all the SVM parameters were set to their defaults. To find the optimal C (defining how much a misclassification increases the cost function) and g (determining the decision boundary of the SVM), a grid-search was performed with two separate steps. First, a grid consisting of 21 steps and 19 steps for the parameters C and g on a logarithmic scale was constructed. The parameter ranges were initially from 10^–5^ to 10^15^ for parameter C and from 10^–15^ to 10^3^ for parameter g. Based on a cross-validation procedure, the SVM classifier with maximum accuracy was selected, and from the corresponding kernel parameters C and g, new parameter ranges were set. After repeatedly running the grid-search with higher resolutions, the grid point with the maximum accuracy of the SVM classifier was chosen, and its corresponding kernel parameters were determined as the optimal values (Additional file 6: Table S1). For the RF classifier, the randomForest function in the R randomForest package [34] was used. The number of random explanatory variables considered as each note was tuned by mtry = number of features, and the number of trees (ntree) was set to 500, its default value.

### Validation and evaluation of model performance

To validate the constructed machine learning models, we performed cross-validation. Both positive (paralogs) and negative (isoforms) datasets were randomized and divided into two parts, each having an equal number of paralog and isoform samples. One-half of the dataset was designated as the training group. The other half was divided into four parts, and each dataset was designated as a testing group. The receiver operating characteristic (ROC) curve was calculated four times based on the vectors of sensitivity and specificity. The values of the area under the curve (AUC) of the ROC curves of the four cross-validation groups were averaged to compare the predictability and stability of the models.

## Results and discussion

### Identification of paralogs and isoforms in RNA-seq data

Because the main objective of this study was to distinguish between paralogs originating from duplication events and isoforms arising from the alternative splicing of a single gene, which recently has become more necessary and demanding with the advent of high-throughput sequencing, we de novo (reference-free) assembled and annotated reference transcriptomes using RNA-seq data. To this end, we strategically selected multiple RNA-seq datasets generated from different species, human, zebrafish, and wheat, which included the brain, ovary, testis, and seed tissues because (1) the brain and testis tissues carry highly abundant alternative splicing events of transcripts [35], (2) the selected human tissues have a high number of expressed genes [20, 36, 37], and (3) the zebrafish and wheat species have complex genomes resulting from ancient whole genome duplication and interspecific hybridization events [22, 38], which can lead to formidable obstacles in distinguishing between paralogs and isoforms (Additional file 7: Table S2). After performing mRNA transcriptome analysis, about 1.6 million transcripts with an average length of 784 bp were de novo assembled, and a total of 275,195 transcripts were uniquely annotated (Additional file 8: Table S3). Using the Ensembl annotation pipeline, each sample contained an average of 11,998 paralogs and 16,998 isoforms (Additional file 9: Table S4).

### Possibly fragmented and misassembled transcripts hamper accurate classification of paralogs and isoforms with sequence alignment-based features

**Fig. 2:**
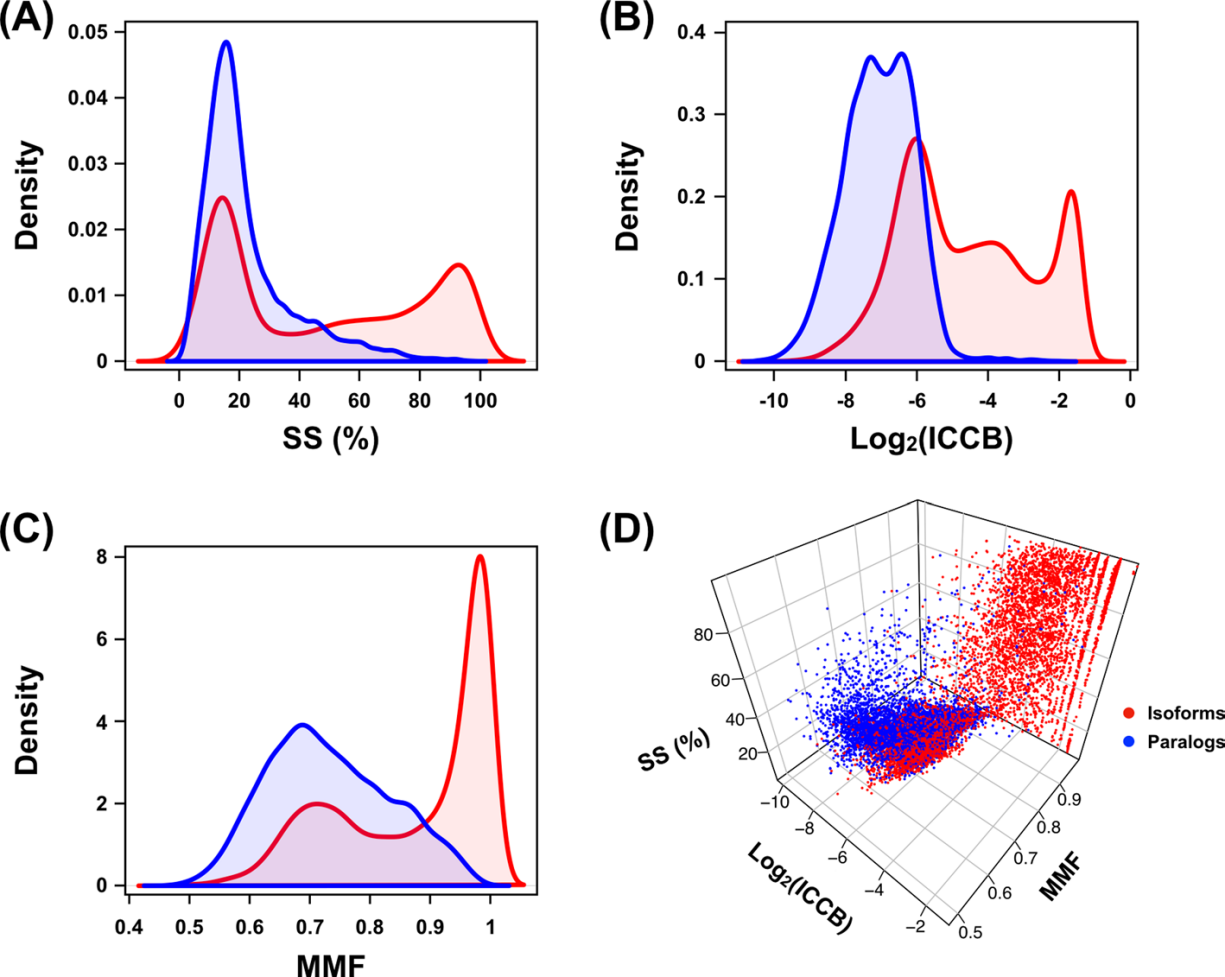
Paralogs and isoforms are poorly classified using pre-existing sequence features. The distributions of SS, ICCB, and MMF are shown in panels **A**, **B**, and **C**, respectively. Panel **D** illustrates all three features at the same time. Samples derived from paralogs and isoforms in the human brain data are shown in blue and red, respectively. The same tests for the other datasets are shown in Additional file 2: Fig. S2–Additional file 5: Fig. S5

Based on various available genomics data, Spitzer et al. [19] proposed three sequence alignment-based features (SS, ICCB, and MMF; Fig. 1) to distinguish between paralogs and isoforms and explored their relevance using full-length cDNAs and EST sequences. To determine whether these features could be applied directly to RNA-seq transcriptome sequences, they were reassessed in our data. A large portion of the matching pairs was well separated into two classes. However, a small but non-negligible number of paralogs and isoforms overlapped with one another and were dispersed with no obvious agglomerate form (Fig. 2). The same patterns were consistently observed regardless of which sample was used for testing (Additional file 2: Fig. S2–Additional file 5: Fig. S5). These results suggest that existing sequence-alignment only features are not sufficient to distinguish between paralogs and isoforms in de novo assembled transcriptome data.

### Minimum cut-off score and gene expression level are considered as potential constitutive features

**Fig. 3:**
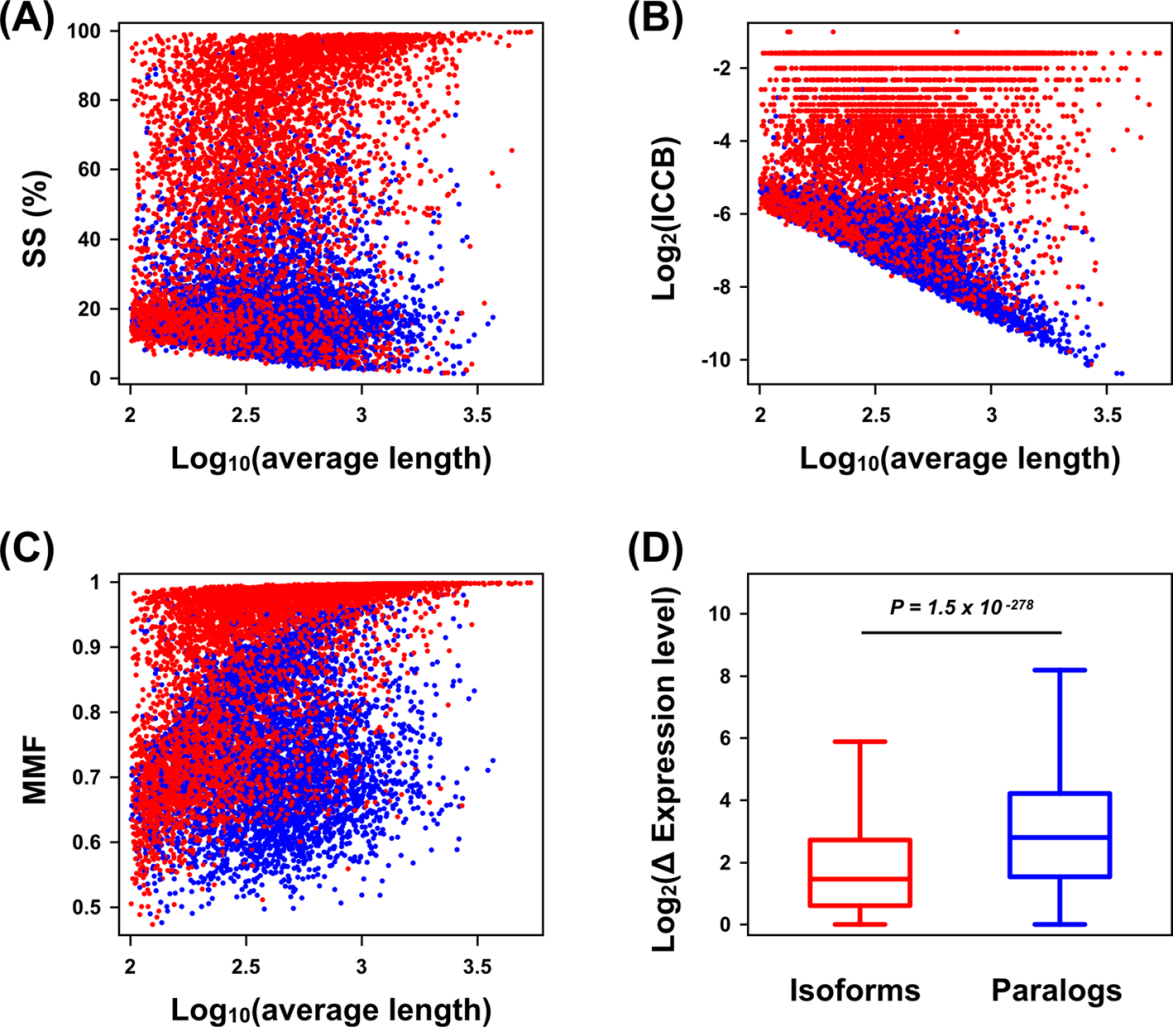
Paralogs and isoforms, especially those with a short gene length, are indistinguishable using the SS, ICCB, and MMF features. **A**–**C** Scatterplots illustrating the combinations of the mean lengths of the pair sequences and each feature. **D** Expression level differences between the isoform and paralog groups. The central line and lower and upper edges of the box indicate the median and 25th and 75th percentiles, respectively. The whiskers extend to the furthest point within 1.5 times the interquartile range (IQR). *P*-values were calculated using the Mann–Whitney U test

A previous study [19] found that the SS, ICCB, and MMF scores were usually higher in isoform pairs than in paralog pairs. Indeed, similar distribution patterns were observed in our data, but a clear classification boundary could not be distinguished. Notably, many of the short sequence pairs were mixed and overlapped (Fig. 3A–C). Thus, we hypothesized that fragmented and misassembled transcripts in RNA-seq transcriptome data with intrinsic methodological issues, including low sequencing accuracy, incomplete gene coverage, and chimerism, represented one of the main causes of the reduction in classification accuracy. To circumvent this issue, we adopted the concept of TZ of sequence alignment for homology modeling to define the sequence similarity limit and used a 20% SS score as the minimum cut-off to be excluded.

According to the classic gene duplication models, a duplicated paralog may result in one of the following: (1) creation of a pseudogene resulting from degenerative mutations (nonfunctionalization) [39], (2) gain of a new function by one of the duplicate genes (neofunctionalization) [8, 40], (3) division of the parental gene’s function between the two duplicate copies after the duplication event (subfunctionalization) [41], or (4) a combination of neofunctionalization and subfunctionalization (subneofunctionalization) [42]. Recently, many genome-wide expression experiments have revealed divergent expression patterns between paralogs [43–45], and these can help to understand the emergence of new gene functions after duplication events [46]. Thus, we predicted that the expression patterns would be distinct between these two structural groups. Indeed, in our data, the expression level differences between paralog pairs were significantly higher than those between isoform pairs (Fig. 3D), indicating its potential as a novel feature.

### Predicting paralogs and isoforms using machine learning models

**Fig. 4:**
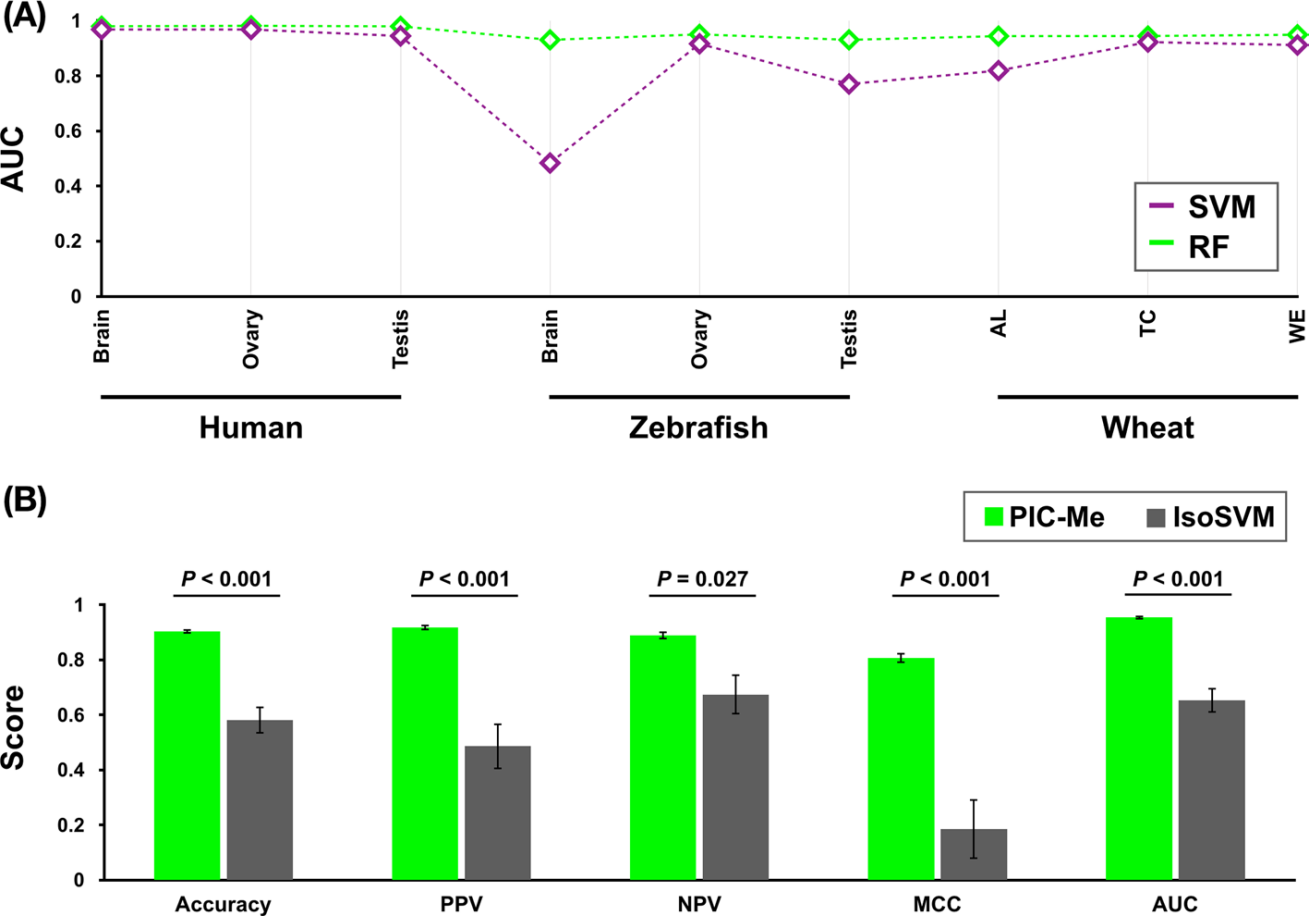
Classifying paralogs and isoforms using machine learning methods. **a** AUC comparison between the SVM and RF models using nine RNA-seq datasets from human, zebrafish, and wheat tissues. AL is the aleurone layer, TC is transfer cells, and WE is whole endosperm. **b** Performance assessment of our method, PIC-me, and a pre-existing method, IsoSVM. Accuracy, positive predicted value (PPV), negative predicted value (NPV), and MCC were calculated as follows: Accuracy = (TP + TN)/(TP + FP + TN + FN), PPV = TP/(TP + FP), NPV = TN/(TN + FN), and MCC = $\frac{TP \times TN-FP \times FN}{\sqrt{(TP+FP)(TP+FN)(TN+FP)(TN+FN)}}$, where TP and TN are true positive and true negative, respectively, and FP and FN are false positive and false negative, respectively. *P*-values were calculated using the Mann–Whitney U test. Error bars indicate the standard error of the mean

To determine whether these five features could be used to classify and differentiate between paralogs and isoforms, we deployed two widely used machine learning models (SVM and RF). With mixed half-split cross-validation (see “Methods”) and using the AUC of the ROC curve, the classification accuracy of our proposed features (TZ and ELD) with the pre-existing features (SS, ICCB, and MMF) was on average 0.826 for SVM and 0.903 for RF. All AUC scores from the RF model were over 0.9 and significantly (*P*-value = 0.002, Wilcoxon paired signed-rank test) higher than those from the SVM model. The lowest AUC score was from the SVM model, 0.484 in zebrafish brain tissue, and this model produced unstable results in the different datasets (Fig. 4A).

To access the performance of our method (implemented using RF, which is hereafter referred to as PIC-Me: Paralogs and Isoforms Classifier based on Machine-learning approaches), we compared PIC-Me with IsoSVM [19], which is a SVM-based classification model that only uses three genomic features (SS, ICCB, and MMF). Based on the five different performance evaluation scores (accuracy, positive predictive value, negative predictive value, Matthews correlation coefficient, and AUC), our proposed PIC-Me method always significantly outperformed the existing IsoSVM method (Fig. 4B).

## Conclusions

To overcome the limitations of conventional full-length cDNA- and EST-based approaches for distinguishing between paralogs and isoforms, which is very challenging when performing the global analysis of the transcriptome of a non-model organism, five distinctive genomic and transcriptomic features were extracted from RNA-seq data, and their use in two machine learning models was examined. Using the RF model with the proposed RNA-seq features, including SS, ICCB, MMF, TZ, and ELD, we developed a machine learning tool, called PIC-Me, and showed that it outperformed an existing classification method. We believe that our tool will be a valuable computational resource for the comparative and evolutionary genomics community [47] and for human disease and cancer biology [48–50].

## Supplementary Information

Additional file 1: Figure S1. Calculation example of three sequence features (SS, ICCB, and MMF).

Additional file 2: Figure S2. Distributions of SS, ICCB, and MMF in two human tissues. Blue and red indicate paralogs and isoforms, respectively.

Additional file 3: Figure S3. Distributions of SS, ICCB, and MMF in three zebrafish tissues. Blue and red indicate paralogs and isoforms, respectively.

Additional file 4: Figure S4. Distributions of SS, ICCB, and MMF in three wheat tissues. Blue and red indicate paralogs and isoforms, respectively. AL is the aleurone layer, TC is transfer cells, and WE is whole endosperm.

Additional file 5: Figure S5. Three-dimensional scatter plots of all three features. (A-C) Human tissues, (D-F) zebrafish tissues, and (G-I) wheat tissues. Blue and red indicate paralogs and isoforms, respectively. AL is the aleurone layer, TC is transfer cells, and WE is whole endosperm.

Additional file 6: Table S1. Optimal parameter C and g.

Additional file 7: Table S2. Statistics of nine public RNA-seq data from human, zebrafish and wheat.

Additional file 8: Table S3. Statistics of *de novo* assembly.

Additional file 9: Table S4. Number of paralogs and isoforms.

## Data Availability

Not applicable.

## Abbreviations

AUC: Area under the curve
CDS: Coding sequence
ELD: Expression level difference
FPKM: Fragment per kilobase of transcript per million fragments mapped
ICCB: Inverse count of consecutive identical or non-identical blocks
MMF: Match-mismatch fraction
PIC-Me: Paralogs and Isoforms Classifier based on Machine-learning approaches
RBF: Radial basis function
RF: Random forest
RNA-seq: RNA sequencing
ROC: Receiver operating characteristic
SS: Sequence similarity
SVM: Support vector machine
TZ: Twilight zone of protein sequence alignment
