# Supplementary figures and images for: PIC-Me: paralogs and isoforms classifier based on machine-learning approaches
Source: BMC Bioinformatics. 2021 Oct 21;22(Suppl 11):311. doi: 10.1186/s12859-021-04229-x (PMC8529730; doi:10.1186/s12859-021-04229-x)

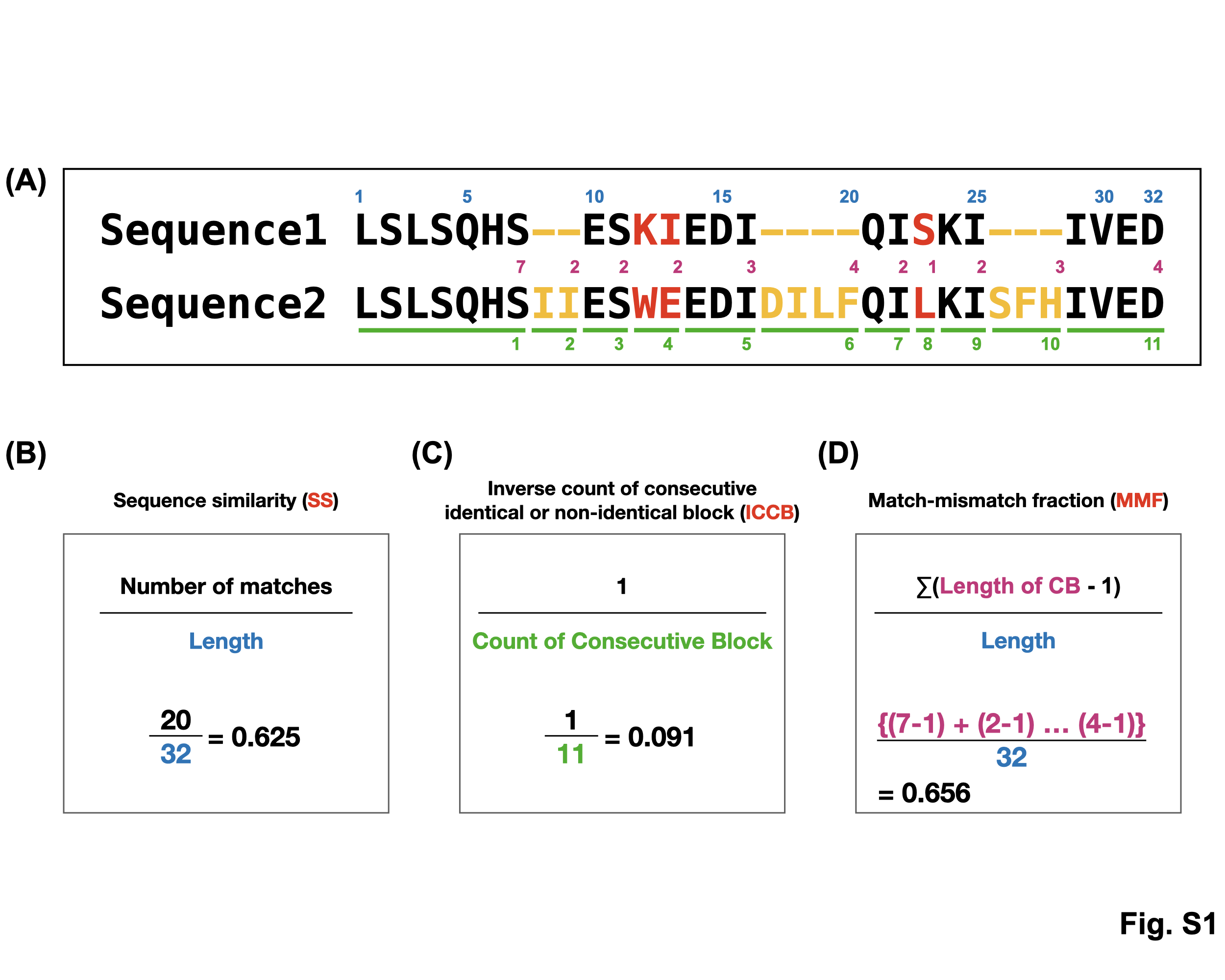

Supplement: Supplementary file 1 — Calculation example of three sequence features (SS, ICCB, and MMF). [file 12859_2021_4229_MOESM1_ESM.tiff]

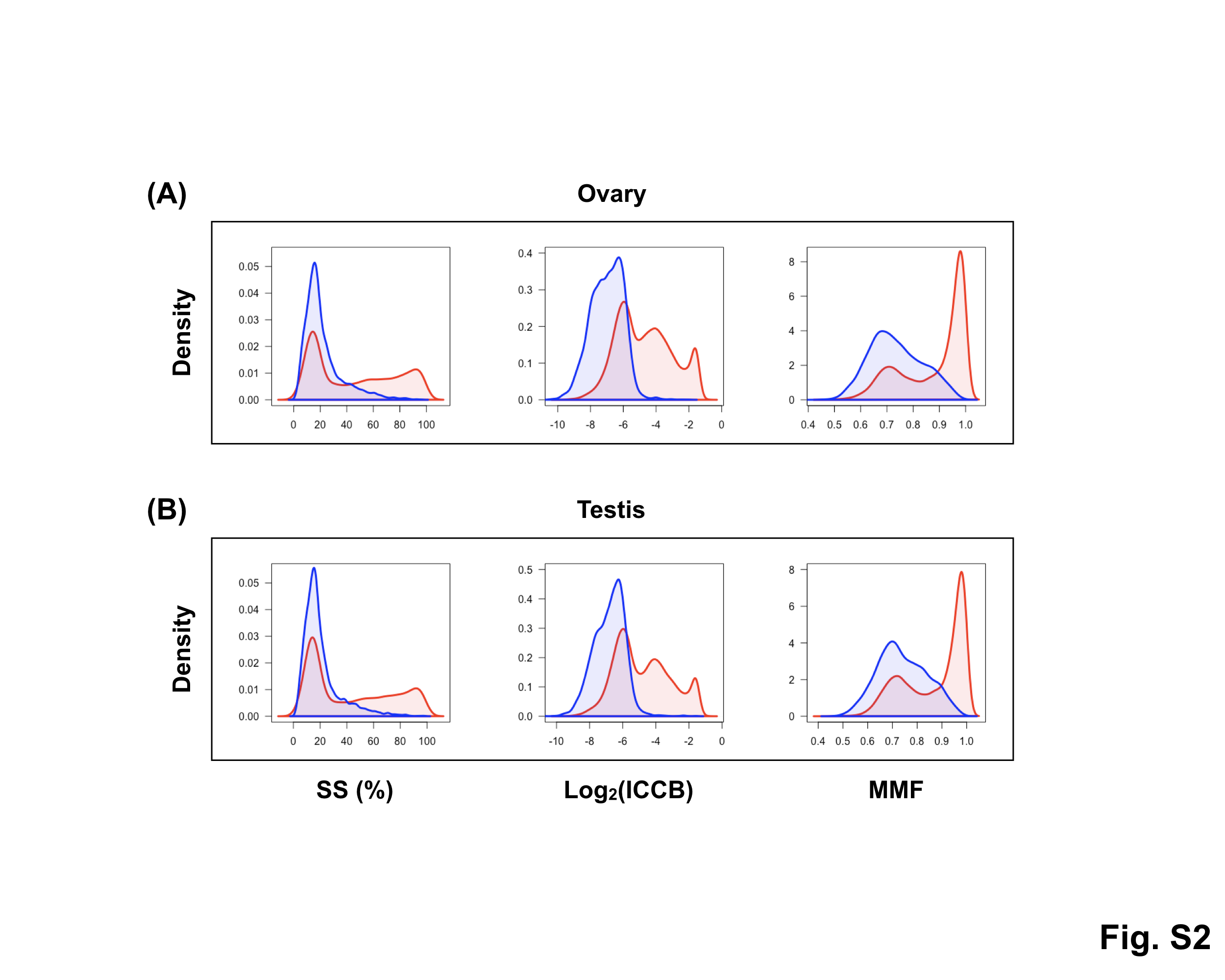

Supplement: Supplementary file 2 — Distributions of SS, ICCB, and MMF in two human tissues. Blue and red indicate paralogs and isoforms, respectively. [file 12859_2021_4229_MOESM2_ESM.tiff]

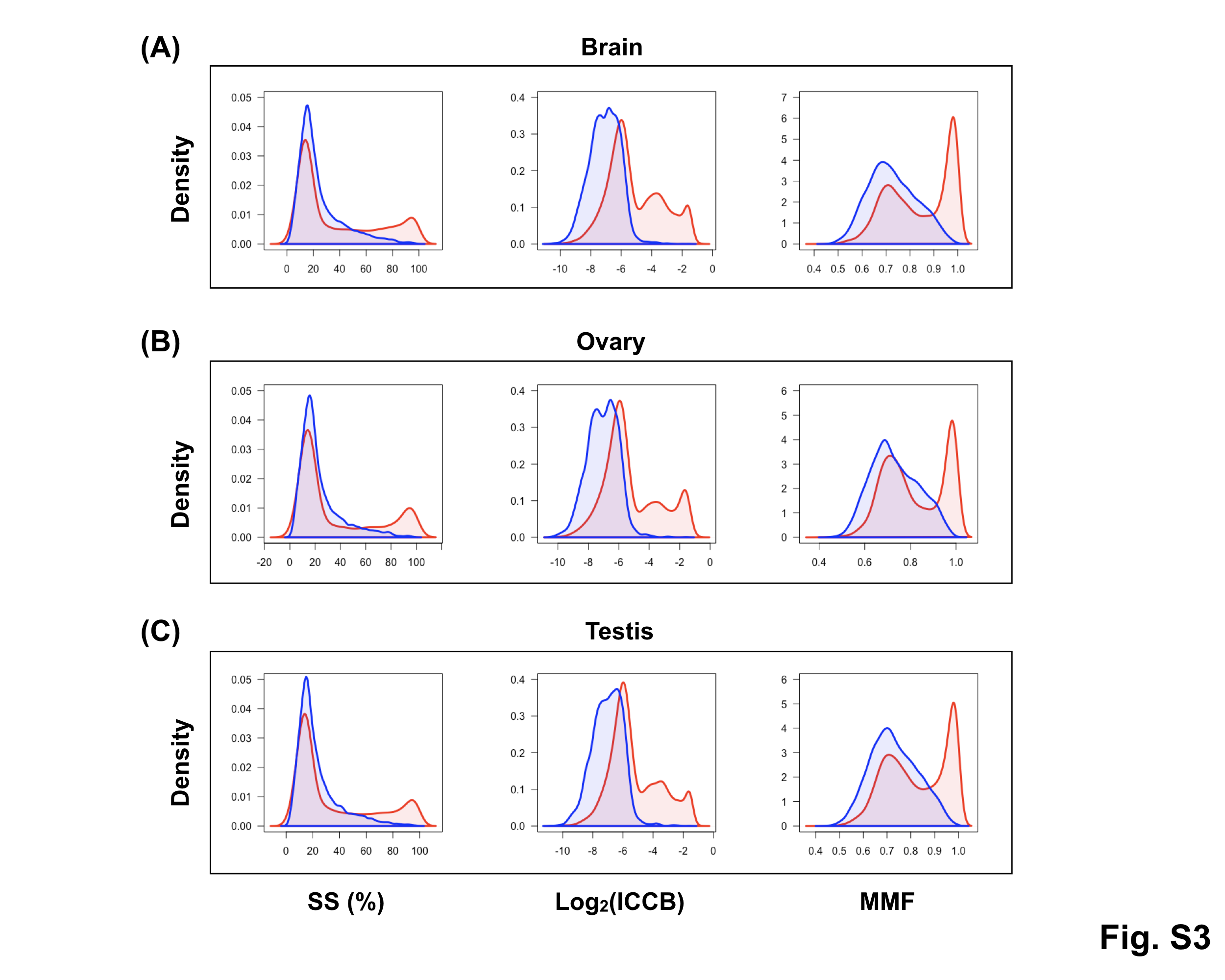

Supplement: Supplementary file 3 — Distributions of SS, ICCB, and MMF in three zebrafish tissues. Blue and red indicate paralogs and isoforms, respectively. [file 12859_2021_4229_MOESM3_ESM.tiff]

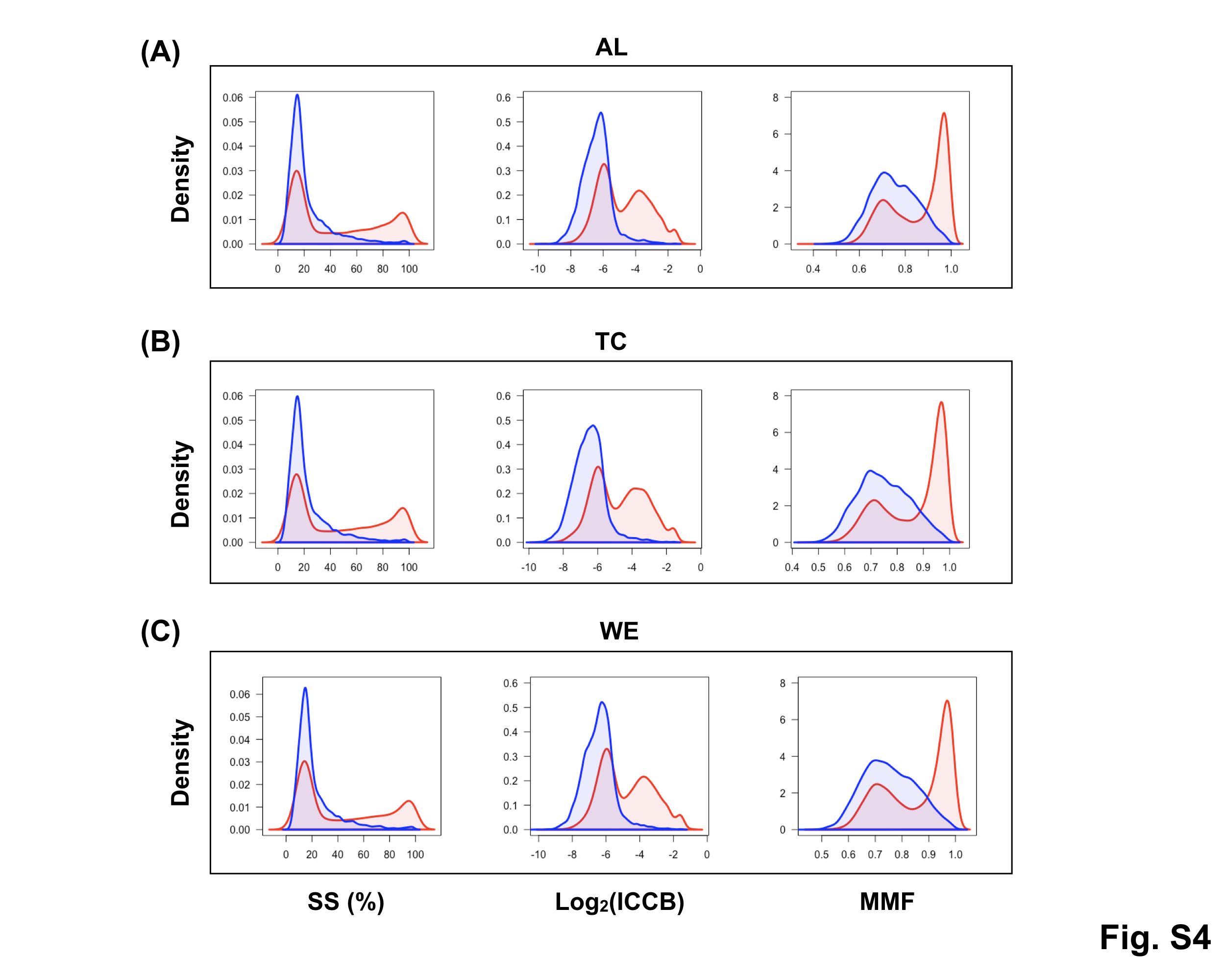

Supplement: Supplementary file 4 — Distributions of SS, ICCB, and MMF in three wheat tissues. Blue and red indicate paralogs and isoforms, respectively. AL is the aleurone layer, TC is transfer cells, and WE is whole endosperm. [file 12859_2021_4229_MOESM4_ESM.tiff]

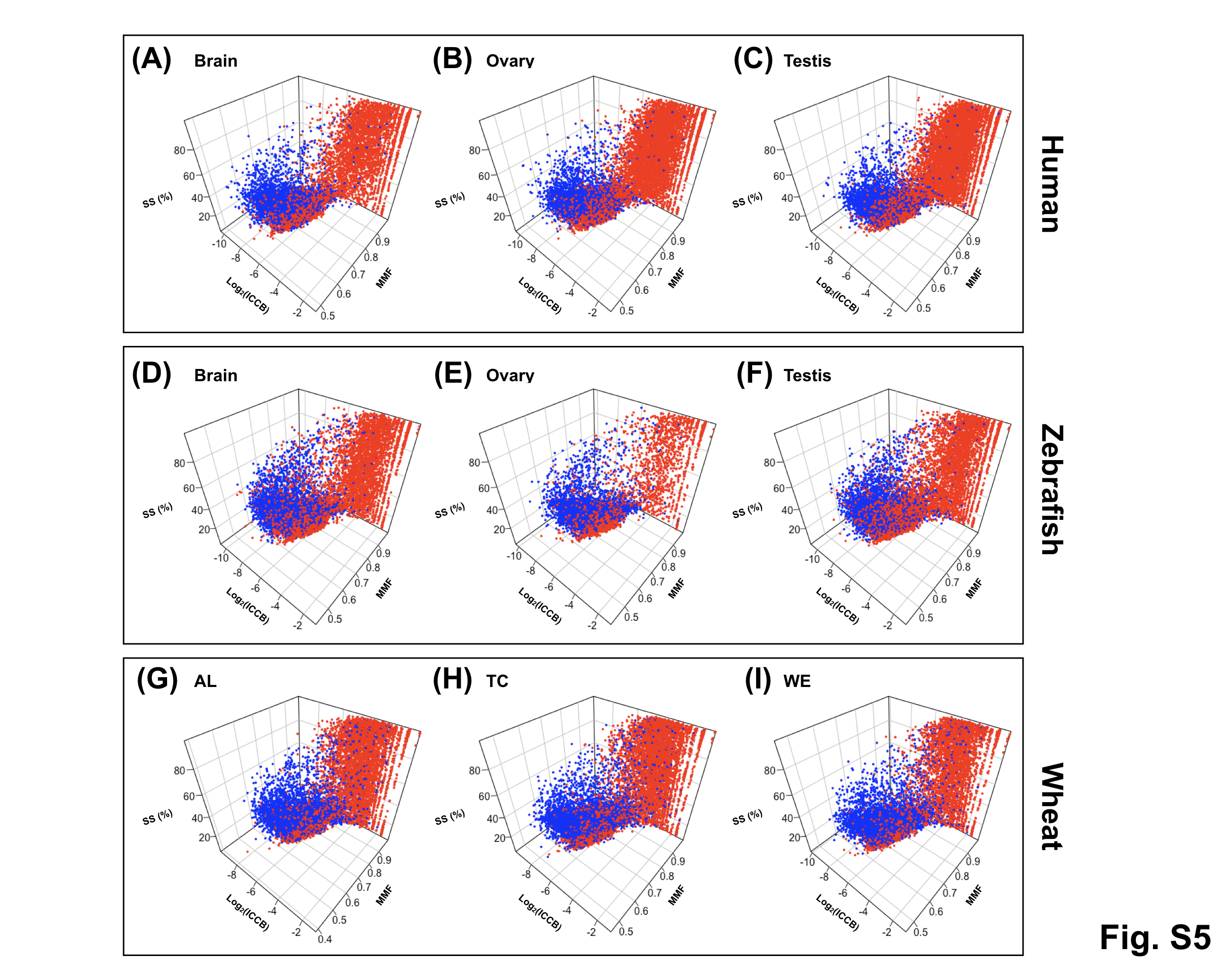

Supplement: Supplementary file 5 — Three-dimensional scatter plots of all three features. (A-C) Human tissues, (D-F) zebrafish tissues, and (G-I) wheat tissues. Blue and red indicate paralogs and isoforms, respectively. AL is the aleurone layer, TC is transfer cells, and WE is whole endosperm. [file 12859_2021_4229_MOESM5_ESM.tiff]
